# Comparison of Trace Elements in High-Molecular-Mass Multiprotein Complex and in Female Milk from Which It Was Obtained

**DOI:** 10.1155/2019/2578975

**Published:** 2019-08-04

**Authors:** Svetlana E. Soboleva, Natalia P. Zaksas, Georgy A. Nevinsky

**Affiliations:** ^1^Institute of Chemical Biology and Fundamental Medicine, Siberian Division of Russian Academy of Sciences, Pr. Akademika Lavrentieva 8, Novosibirsk 630090, Russia; ^2^Nikolaev Institute of Inorganic Chemistry, Siberian Division of Russian Academy of Sciences, Pr. Akademika Lavrentieva 3, Novosibirsk 630090, Russia

## Abstract

**Background:**

Many biological processes are performed by different protein complexes. During the association of proteins and enzymes forming specific complexes, the latter can include ions of various metal ions, which may be important for their formation and biological function.

**Objective of the Studies:**

However, to date in the literature there are no data on metal ions that are part of any protein complexes.

**Methods:**

A very stable multiprotein complex (~1000±100 kDa) was separated from other proteins of nine samples of female milk by gel filtration on Sepharose 4B. The content of microelements in the stable multiprotein complex and milk was analyzed using two-jet plasma atomic emission spectrometry.

**Results:**

The content of different elements in milk on average decreased in the order: Ca>P>Mg>Al≥Zn≥Fe>Cu >B (0.76–3500 *μ*g/g of dry milk powder), while the content of some elements was very low (Sr>Mn>Cr>Ba>Pb>Ag>Ni>Cd, <0.03–0.5 *μ*g/g). The content of eight elements in stable multiprotein complex was 1.2-9.6-fold higher than in milk and increased in the order: Ca≈Mg<P<Al<Fe<Pb<Ba<Cr<Cd<Zn, while content of SPC eight metals was 12.3-110-fold higher: Cu (12.3)>B (19.7)>Ag (28.7)>Ni (38)≥Sr (110).

**Conclusions:**

The analysis of the relative content of sixteen elements in human milk and oligomeric complexes of proteins was performed for the first time. Data on the content of metals indicate that during the formation of protein which associates the increase in the content of metal ions bound with proteins of the complex can occur. Such metal ions can be important for the formation and biological function of protein complexes.

## 1. Introduction

Microelements play different important roles in many biological processes [1–10]. They participate in the transport of gases and nutrients, support acid-base balance, temperature, maternal and child mental health, homeostasis of the human organisms, the functioning of enzymes, protein and DNA syntheses, cytoskeleton activation, etc.

Today there are many methods for elemental analysis of different biological samples. Atomic absorption spectrometry (AAS), inductively coupled plasma atomic emission (ICP-AES), and mass spectrometry are usually used for analysis of blood and animal tissues [11, 12]. These methods generally require matrix destruction with concentrated acids.

**Figure 1:**
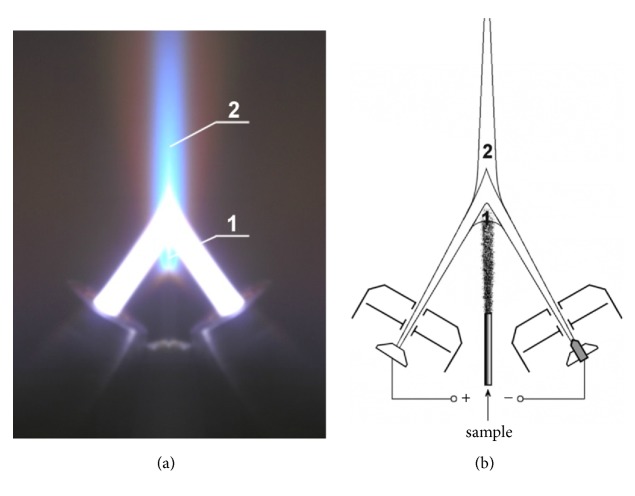
Plasma torch (a); electrode unit and analytical regions of the plasma flow (b): 1, before the jet confluence; 2, after the jet confluence.

In the present study, two-jet plasma atomic emission spectrometry (TJP-AES) was applied. It was developed in the mid-1970s [13]. The TJP is a direct current (dc) plasma that differs from dc plasmas described [14, 15] and an ICP by a high power (up to 15 kW), which allows analysis of powdered samples without sample dissolution. The plasma torch photograph and scheme of electrode unit are presented in Figure 1.

First, the TJP-AES was used for direct analysis of sparingly soluble geological samples [16]. The possibilities of the method for analyzing biological samples were shown by simultaneous analysis of different elements in dried and finely powdered bovine liver [17]. The estimation of Al, Ca, Cu, Fe, Mg, Mn, Mo, P, Si, Zn, Fe, Mn, and Mo concentrations in liver was performed by direct technique. The use of the procedure of carbonization allowed determining the low concentrations of Ag, Cd, Co, Cr, Pb, and Ni in the liver. Then the main essential (Fe, P, Ca, Mg, Zn, and Cu) and other elements were estimated in different freeze-dried and carefully mashed samples of organs [18], whole blood [19], and bone [20] of animals. The possibility of small amount samples (several mg) analysis allows the use of TJP-AES in the case of biomedical experiments with small animals [21] including in homogeneous IgGs [22]. In contrast to plasmas, IgGs did not contain a detectable amount of Ti, Sr, Mo, Ag, and Cr. The relative amount of various metals bound to IgGs in average decreased in the following order: Fe ≥ Pb ≥ Zn ≥ Cu ≥ Al ≥ Ca ≥ Ni ≥ Mn > Co ≥ Mg [22]. It was shown in several articles that electrophoretically, and immunologically homogeneous polyclonal IgGs from sera of healthy volunteers and experimental mice could not catalyze different chemical reactions [23–27], but they catalyze different chemical reactions after addition of external metal ions: peroxidase (H_2_O_2_-dependent) and oxidoreductase (H_2_O_2_-independent) oxidation of substrates, hydrolysis of DNA, RNA, proteins, and peptides [23–27].

Human breast milk is much more than nutrient system promoting neonatal growth. The milk contains significant amounts of many bioactive compounds including metal ions, which are integral parts of the infant's intestinal physiology and protectors from viral and bacterial infections [28–30]. The toxic effects of EDTA on breast milk include cell loss, disruption of milk fat globule membrane and subsequent release of membrane-bound protein, free fatty acids, and reduction in pH [31]. Therefore, identification and characterization of components of human milk including metal ions is an important step in understanding milk function.

Five samples of human milk were deproteinized, preconcentrated differently with 1% 8-hydroxyquinoline and 1% ammonium pyrrolidine-dithiocarbamate, and extracted with methyl-isobutylketone. Activated carbon powder in HNO_3_ was also used. The obtained extracts were analyzed using atomic absorption spectrometry for some metal ions [32].

It has been proposed that many biological processes are performed by protein complexes [33, 34]. Using different methods, we have recently shown the existence of very stable multiprotein complexes (SPCs) with high-molecular masses (~1000±100 kDa) in human milk [35] and placentas [36]. According to SDS-PAGE, these SPCs contain several different major and minor proteins with high, moderate, and low molecular masses [35, 36]. The complexes dissociate only in the presence of 8 M urea supplemented with 1.0 M NaCl and EDTA. SPCs of human milk possess metal-dependent DNase activity [35].

Very stable complexes of proteins are likely to exist in various biological liquids. However, in the literature, there is as yet no data on possible type of metal ions and their role in the formation and stabilization of the protein complex as well as their functioning and manifesting catalytic activities.

Therefore, in this article, using TJP-AES we have analyzed for the first time the relative content of different metals in preparations of stable protein complex and in human milk from which the complex was obtained.

## 2. Materials and Methods

### 2.1. Reagents

Reagents including Sepharose 4B used in this work were obtained mainly from Sigma (St. Louis, MO, USA) and Merck (Darmstadt, Germany). The milk sampling protocol conformed by the ethics committee of Novosibirsk State Medical University (Novosibirsk, Russia). This study was approved including written consent of healthy mothers to present their milk for scientific purposes in accordance with Helsinki ethics committee guidelines.

### 2.2. Purification and Analysis of Milk Multiprotein Complex

Milk of nine healthy women (200–400 mL) was collected at 20-22 days after the beginning of lactation under sterile conditions using a breast pump and used within 1–3 h after the collection.

All procedures for isolating the protein complex were carried out using milliQ water, different solutions, and buffers that were missed through columns with Chelex 100 (Sigma) to remove possible traces of metal ions. All used utensils including Eppendorf plastic tubes were first washed three times with a solution of 0.1 M EDTA and then 20 times with milliQ water. Dialysis bags before use were also treated with a solution of EDTA and then with milliQ water.

To separate out the fats, lipids, and the cell pellet before gel filtration the milk samples were centrifuged (50 min, 8000×*g;* Beckman Coulter Avanti-J-301 centrifuge; Beckman Coulter, Brea, CA, USA ) in tubes pretreated with EDTA solution and milliQ water (Millipore Simplicity from Millipore, Burlington, MA, USA). Then, the resulting skimmed milk containing no cells were passed through Sephadex G-75 column (20 mL; Sigma) equilibrated with buffer A (20 mM Tris-HCl, pH 7.5 containing 0.15 M NaCl) for additional separation of the fat-lipid fraction and twice dialyzed against milliQ water.

Finally, the skimmed milk preparations were concentrated approximately tenfold in a dialysis bag exposed to constant airflow at 4°C and subjected gel filtration on a Sepharose 4B column (50 mL) equilibrated in A buffer. To obtain necessary amount of SPCs, nine concentrated skimmed milk preparations (1 mL) were subjected to gel filtration several times. The proteins were eluted using the same buffer, and eluate was monitored by absorbance at 280 nm. Fractions (2-3 mL) were collected using Eppendorf plastic tubes, three times dialyzed for removing of NaCl against milliQ water for 16 hours at 4°C. The concentration of the SPCs in the final solutions was measured using the Bradford assay with a bovine serum albumin standard as in [37]. Equal quantities of SPCs from the nine milk preparations were mixed (SPC_mix_). The resulting solution was lyophilized and the fine powders (5.5 mg of SPC_mix_ per one of three independent analyses) were used to estimate the content of different metals by TJP-AES as described below using a multielement photodiode analyzer of emission spectra produced by *«*VMK Optoelektronika*»* (Novosibirsk, Russia).

### 2.3. Analysis of SPC Stability

The stability of ~1000 kDa SPC_mix_ was analyzed by light scattering approach (LS) and gel filtration as in [35, 36]. The reaction mixtures in LS experiments contained SPC_mix_ (0.5 mg/mL) in buffer A (150 мМ NaCl, 20 мМ  Трис-HCl, pH 7.5). All measurements were performed at 20°C using monochromatic laser coherent light (430 nm) and special equipment constructed by Tyzikov F. (Institute of Catalysis of RAS, Novosibirsk, Russia) [25, 36] LS was measured by a standard square optical quartz cuvettes (50×50 mm, wall thickness 1 mm). The LS data were corrected taking into account internal sample absorption and background scattering as in [35, 36]. Several different compounds were added to the reaction mixtures in different combinations: 1 M NaCl, 1-2 M MgCl_2,_ 8 M urea, and 10 mM DTT. Time dependent LS was measured. The SPC_mix_ before and after its treatment with different compounds was analyzed using gel filtration on Sepharose 4B as described above according to [35, 36].

### 2.4. Metal Content Analysis

Individual preparations of nine samples of milk (30 mL) from nine donors were thoroughly dried by lyophilization. One milliliter of different milk contains 132 ± 9.3 mg of dried powder. Then the dry mixture was thoroughly rubbed, and the fine powder was additionally dried. Samples of final powder (6-7 mg) were used for analysis by TJP-AES.

The TJP-AES analysis was performed using the following conditions: current strength – 80-85 A, plasma gas – 4 l/ min, carrier gas – 0.7 l/min, angle between jets – 60°, and analytical region – 4-5 mm lower than the point of the jet confluence. A diffraction spectrograph with a 2400 lines/mm grating covering two spectral ranges (185–350 and 385-470 nm) was used. Spectrum registration was performed using a multielement photodiode analyzer of emission spectra produced by *«*VMK Optoelektronika*»* (Novosibirsk, Russia). Graphite powder containing 15 wt. % NaCl with the impurity concentration range of 0.01–500 *μ*g/g was used to obtain calibration curves. These samples were from Russian State Certified Reference Materials of graphite powder with different composition of impurities (SOG-24, SOG-37, and SOG-21 containing 24, 37, and 21 elements, respectively; Ural State Technical University, Yekaterinburg, Russia) in a clean room designed for manipulation with high-purity samples. The calibration samples are stable for at least a year. The final mass percentage of each metal was estimated from the difference between the corresponding experimental and control powder samples. The data are presented as micrograms of chemical element per gram of every powder and then recalculated as mg of element per 1 liter of the plasma. The relative content of different elements in SPC_mix_ was performed by similar way using its fine powders (5.5 mg of SPC_mix_ per one of three independent analyses).

### 2.5. Statistical Analysis

The average values of all parameters analyzed (mean ± SD) were estimated using three independent assays for each sample of milk and SPC_mix_. The relative standard deviation for every sample analyzed was within 5-7 %. The Shapiro-Wilk criterion test was used to check the normality of the values distribution. Many of the sample sets were not fit the Gaussian distribution. For such value sets median (M) and interquartile ranges (IQR) were additionally estimated.

## 3. Results

### 3.1. Preparation of Multiprotein Complex

**Figure 2:**
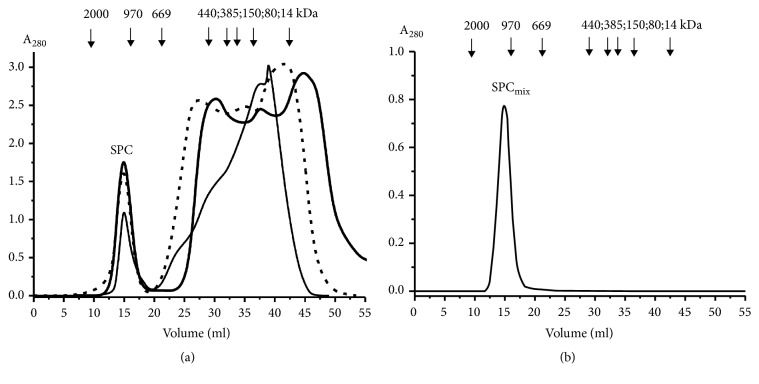
Isolation of milk very stable protein complex (SPC) by gel filtration on Sepharose 4B (50 mL) of fresh human milk (1 mL) of three donors. (a) Rechromatography of equimolar mixture of complexes from nine milk preparations (SPC_mix_) on Sepharose 4B. Lines of three profiles of Panel (a) and one profile of Panel (b) correspond to absorbance at 280 nm (A_280_).

**Figure 3:**
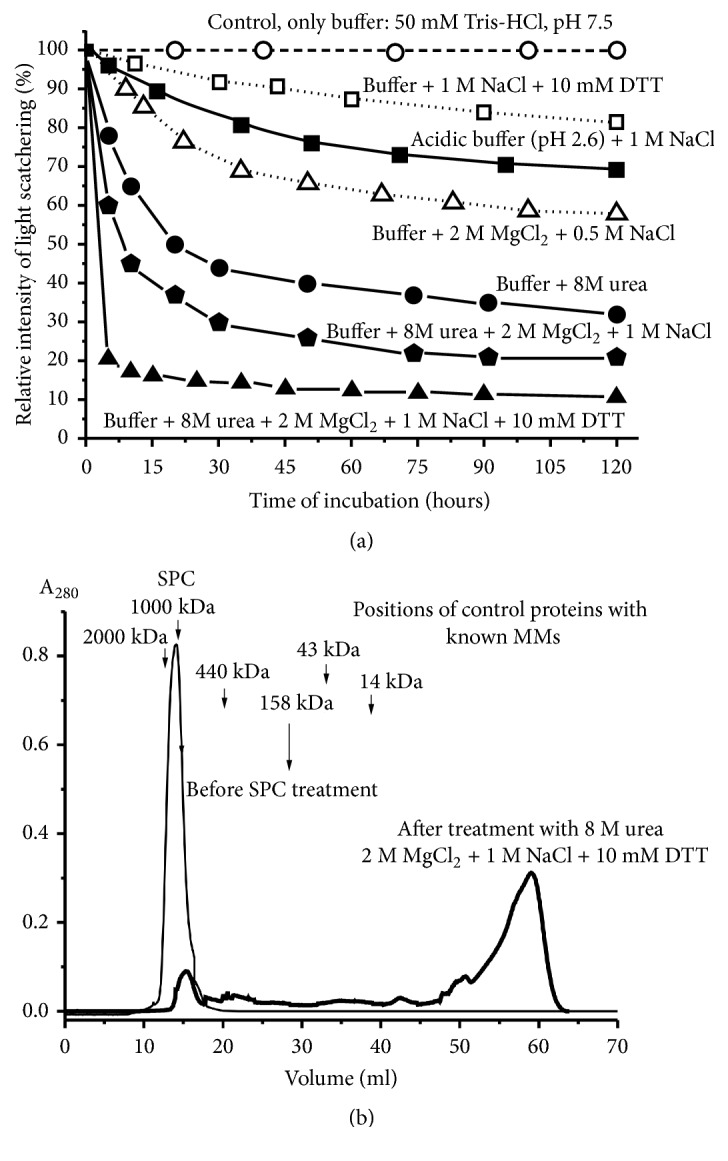
Typical examples of the time courses in changes of the relative light scattering (LS) intensity of the SPC_mix_ (0.5 mg/mL; mixture of the complexes from nine milk preparations) in different conditions (a). The relative maximal light scattering at the experiment zero time was taken for 100 %. Gel filtration on a Superdex 200 of the SPC_mix_ after its incubation for 120 h in buffer containing 8 M urea +2 M MgCl_2_+ 1 M NaCl and 10 mM DTT: (—) absorbance at 280 nm (A_280_) (b). For details, see Materials and Methods.

For preparation of SPCs nine samples of individual fresh milk were subjected to gel filtration on Sepharose 4B column equilibrated in buffer A (Figure 2(a)) as in [35, 36]. Figure 2(a) demonstrates three typical profiles of the gel filtration. One can see that one symmetrical protein peak of high-molecular mass (~1000 ± 100 kDa) is well separated from peaks of other proteins. During repeated gel filtration, only one peak of SPC_mix_ was detected (Figure 2(b)), the position of which corresponded to its position at SPC isolation (Figure 2(a)), and there are no other peaks of its possible protein fragments. It was previously shown that the multiprotein SPC from breast milk is very stable even under very rigid conditions [35]. In this work, we confirmed previously obtained data [35] concerning the complex stability using new SPC_mix_ preparation from new samples of milk (Figure 3). It is known that at high concentration NaCl and MgCl_2_ efficiently dissociate different proteins complexes. According to the LS data, SPC_mix_ was very weakly destroyed in the presence of 1 M NaCl even together with 10 mM DTT or acidic buffer (pH 2.6) containing 1 M NaCl in the conditions of antibody-antigen complex destroying (Figure 3(a)). However, SPC_mix_ was efficiently destroying in buffer containing 8 M urea and even better in the buffer containing 8 M urea, 1 M NaCl, and 2 M MgCl_2_ (Figure 3(a)). Urea usually mainly breaks down hydrogen bonds and less electrostatic interactions between the molecules. Therefore hydrogen bonds between various proteins most probably could play an important role in SPC stabilization. Since NaCl and MgCl_2_ separately and in the presence of urea also increase the SPC_mix_ dissociation (Figure 3(a)), it can be assumed that some of contacts between molecules of proteins are electrostatic. It should be emphasized that addition of DTT to this mixture significantly stimulates destroying of the SPC_mix_. This points to the fact that some molecules of the SPC_mix_ proteins with low, average, and high, molecular masses can be bound by covalent disulfide S-S bonds. From our point of view such a very stable complex cannot be result of a random association of different milk proteins.

After the maximal destruction of the SPC_mix_ using the urea buffer containing 10 mM DTT, the final mixture was subjected to gel filtration on the column with Sepharose 4B. Two small peaks of no completely dissociated SPC_mix_ demonstrated MMs remarkably lower (400-700 kDa) than the initial intact complex (Figure 3(b)). However, on the whole, there was a distribution of proteins (A_280_) with different MMs across the entire chromatography profile.

Thus, in this work, we confirmed the exceptional stability of new preparation of SPC from breast milk. The resulting solution of equimolar amounts of the complexes from nine samples of different milk (SPC_mix_) was thoroughly dried by lyophilization and used for analysis of the content of various elements (Table 1).

### 3.2. Analysis of Milk SPC Metals and Other Elements

To analyze elements of a very stable protein complex, we have used SPC_mix_ obtained by gel filtration on Sepharose 4B as in [35, 36]. The resulting solution of equimolar amounts of the SPCs from nine samples of milk (SPC_mix_) was thoroughly dried by lyophilization and used for analysis of the content of various elements using the TJP-AES method (Table 1). The relative content of different elements in SPC_mix_ decreased in the order: Ca >P > Zn >Mg >Al ≥Fe > Cu >Ni≥B ≥Sr>Mn≥Cr≥Ba≥Pb>Cd≥Ag (Table 1).

### 3.3. Estimation of the Relative Content of Different Elements in Human Milk

It was interesting how different the content of various elements in the SPC_mix_ and in the milk preparations used for its isolation. In contrast to SPC_mix_, the quantity of each of the lyophilized milk preparations was enough for their individual analysis using the TJP-AES method allowing determination of many elements simultaneously (Table 2). The relative content of different elements varied differently depending on the element analyzed between the individual preparations.

The content of different elements in milk on average decreased in the order: Ca > P>Mg >Al≥Zn≥ Fe > Cu>B (Table 2). The content of several elements (Ni, Ag, Sr, Ba, Pb, Cd, Cr, and Mn) in the milk was very low, < 0.03-0.5 *μ*g/g of powder.

## 4. Discussion

In this paper, we have isolated a high-molecular multiprotein complex from female milk and showed its extreme stability. We compared in this paper the relative content of trace elements in the composition of SPC_mix_ and milk preparations from which this complex was isolated.

Literature data on the analysis of elements of breast milk are very different and contradictory. For example, the relative average concentrations of different elements were analyzed using milk samples collected in Argentina, Namibia, Poland, and United States [38]. It was shown that the concentrations of different elements estimated by ICP-MS in human milk may be very variable among the populations. The maximum difference 4.8-fold in the average content of manganese is found in milk of Argentina (7.6 mg/l) and Poland (1.6 mg/l) women [38]. As noted by the authors [38], one of the reasons for such differences may be the individual characteristics of each woman as well as changing diets and environments worldwide. The authors of [38] also analyze the literature data on the analysis of trace elements in the milk of women from different countries using various approaches. The relative concentrations of elements depend not only on the diverse in populations but also on the approaches used for their estimation. For example, the average iron concentration in the milk of Japanese women found using ICP-AES (2.5 mg/l) is about 16 times higher than when using the AAS approach (0.16 mg/l) ([38–40]).

In addition, the relative content of certain metal ions (Ca, Mg, Zn, Fe, Cu, Cr, and Mn) in human milk was previously evaluated using two approaches using 8-hydroxyquinoline (method 1) and ammonium pyrrolidine-dithiocarbamate (method 2) [32] (Table 3). One can see that the relative content of all metals is significantly varied for various milk preparations. In addition, the use of these two approaches leads to very significant differences in the estimation of the relative content most of the metals (Table 3). For example, the concentration of calcium ions in the case of these two methods (8.4–688.9 and 0.90–12.7 mg/L) differs 9.3-54-fold (Table 3).

We compared the data obtained by us with the published results [32, 38] on the evaluation of the content of some metals in human milk (Table 3). Taking into account the essential difference in the content of all elements in different milk preparations, some our data agree to some extent with data of [38], while other with finding of [32]. However, in this article, we estimated the content of the elements only in nine milk preparations that were used to purification of SPC_mix_. As it was shown in several articles [16–22] the analysis of control and experimental samples using TJP-AES leads to well reproducible results. The difference in the values obtained by TJP-AES in comparison with the known content of elements in the control samples does not exceed 3-7 %. Moreover, TJP-AES allows simultaneous evaluation of the content of a large number of elements using the same sample. However, limitations due to restricted number of milk samples prevent us from drawing robust conclusions concerning a possible difference in the content of trace elements in milk of females living in Russia and other countries.

As it was shown previously, the SPCs from different milk contain approximately: LF (60 %) LA (30 %), casein (3-5 %), HSA (3-5 %), and immunoglobulins (3-5 %) [35]. LF (76–80 kDa) consists of two lobes, each of which contains one hemoporphyrin structure binding Fe^3+^ ion [41]. The elements estimated in this study are involved in many biochemical processes. It is interesting that SPC_mix_ contains some elements in significantly higher concentration than human milk (Table 1). Approximately 10-15% of lactoferrin molecules of human milk contain iron ions. Therefore, it is not surprising that the relative content of iron ions in the SPC_mix_ is approximately in 3.8-fold higher than in the milk (Table 1). In addition, LF can bind other various different metal ions (Мg^2+^, Ca^2+^, Cu^2+^ Zn^2+^, etc.) [35]. LA binds specifically to Ca^2+^in molar 1:1 ratio [42]. However, it can interact with many other different metal ions. HSA has three [43] or four [44] binding sites interacting with many different metal ions: Ca^2+^, Mg^2+^, Mn^2+^, Co^2+^/Co^3+^, Al^3+^, Ni^2+^, Zn^2+^, Cd^2+^, Cu^1+^/Cu^2+^, Pt^2+^, Au^1+^/Au^2+^, Hg^2+^, and Tb^3+^. Immunoglobulins in milk and blood are also associated with a large number of very different metal ions [45]. Casein is phosphoprotein, which exists in milk as a calcium salt, but it can also bind other metal ions [46]. Thus, these proteins can bind various milk metals and increase their relative amount in the complex. Therefore, it was not surprising that the sum of all metal ions in 1 g of SPC is approximately 1.6-fold higher than in 1 g of milk powder (Table 1).

Concentration of some elements (Ca, Zn, Mg, Fe, Cu, Mn, Cr, and Pb) of human milk was estimated in several articles using different methods (Table 3). Using the TJP-AES, we carry out for the first time a quantitative estimation of the content in human milk of P, Al, Ni, and B (Table 3). The peaks of some elements (Sr, Ba, Cd, and Ag) in the spectra were reliable, but were sufficient only for approximate estimation of their content (Tables 2 and 3). At the same time, the content of all these elements in the preparation of the SPC_mix_ was increased and quantitatively reliably tested (Table 1).

Interestingly, the efficiency of the accumulation of metal ions, which are contained in milk in an increased concentration, by the proteins of the SPC_mix_, is very different and decreased in the following order (-fold): B (19.7) > Cu (12.3) > Zn (9.6) > Fe (3.8) > Al (3.0) ≥ P (2.6) > Ca (1.2) ≈ Mg (1.2) (Table 1). An even more unexpected situation is the very strong increase in the relative content of ions in the very stable complex observed for metals that contained in milk in a relatively low concentration (-fold): Sr (>110) > Mn (>82) > Ni (> 38) > Ag (>28.7) > Cd (>9.0) > Cr (6.2) > Ba (>5.8) > Pb (>4.0) (Table 2).

Interestingly, the SPC_mix_ contains a relatively large amount of phosphorus (2.5 mg/g, Table 2). It is due to the fact that, in addition to proteins, this complex contains relatively short DNA and RNA molecules, which can also interact with ions of different metals [35]. In addition, casein is known as phosphoprotein [46], which can also contribute to the content of phosphorus in milk and SPC_mix_. It is reasonable to suggest that some of the metal ions can be bound with the phosphate groups of DNA and RNA, while calcium and other ions can be bound with casein and its phosphate groups.

The metal ions revealed can be bound with the specific centers of proteins chelating different metal ions. For example, 1 g of SPC_mix_ contains approximately 0.06 *μ*moles of LF, but 1.9 *μ*moles of Fe^2+^ ions. In the calculation for two LF centers and the protein saturation, it can content 0.12 *μ*moles of Fe. However, only 10-15% of milk LF contains Fe ions [35]. It means that other proteins of SPC_mix_ can also bind Fe ions.

An increase in the relative content of several metals, which are contained in milk in an increased concentration, in the SPC_mix_ compared with the milk seems quite expected, since these metal ions (Mg, Ca^2+^, Mn^2+^, Cu^2+^, Ni^2+^, and Zn^2+^) are more often involved in the function of different proteins and enzymes. One of the proteins possessing a specific centers having increased affinity Zn^2+^ and Cu^2+^ ions is HSA [43, 44]. Active centers of antibodies with catalytic activities (abzymes) hydrolyzing DNA, peptides, and proteins as well as catalyzing other different reactions most often use cofactors Ca^2+^, Mg^2+^, Mn^2+^, Cu^2+^, Ni^2+^, Fe^2+^, and Zn^2+^ [22–27]. Human lactoferrin is metal-dependent DNase activating by Ca^2+^, Mg^2+^, and Mn^2+^ ions [47, 48]. However, the relative increase in the relative content of these metals (Ca, Mg, Fe) in SPC_mix_ compared to milk is relatively small, only 1.2-3.8-fold (Table 1). Of this group of metals, the exception is zinc (9.6), copper (12.3), and nickel (>38).

1 g of SPC corresponds approximately to 0.08 *μ*moles of HSA, but 5.2 *μ*moles of Zn^2+^ and 0.77 *μ*moles of Cu^2+^ions. Most likely, that in the binding of Zn^2+^ and other metal ions, several other proteins of the SPC can also participate.

Interestingly, the increased content in the SPC_mix_ of some metal ion in comparison with milk is somewhat unexpected (-fold): Sr (>110) > Mn (>82) > Ni (>38) >Ag (>28.7) > Cd (>9.0) > Cr (>6.2) > Ba (>5.8) > Pb (>4.0) (Table 1). However, it is known that some centers of proteins having an increased affinity for specific metals are not very specific and can bind a wide variety of other metal ions with a lower affinity. For example, two centers of HSA specific for zinc and copper as well as the third multimetal binding site effectively bind many different metal ions including Al^3+^, Cd^2+^, Pt^2+^, Au^1+^/Au^2+^, Hg^2+^, and Tb^3+^ [43, 44].

As noted above, the destruction of a very stable SPC requires the presence of EDTA [35]. This may indicate that certain metal ions can play an important role in the metal-dependent electrostatic interaction between different proteins of the complex. In addition, it cannot be ruled out that additional centers capable of chelating different metal ions can be formed at the junction of protein molecules in the complex. These new chelating centers can in principle have a different specificity in the binding of metal ions comparing with the individual proteins of the SPCs. In addition, one cannot exclude that metal ions can enter the cavity (or holes) between molecules of the SPC during proteins association.

The main component that destroys the SPC_mix_ is 8 M urea, which indicates that SPC proteins form mainly hydrogen bond to each other [35]. Acceleration of the SPC dissociation after the addition of 1 M NaCl indicates the formation of electrostatic contacts between the SPC proteins. The SPC does not lose metal ions during its isolation and subsequent dialysis. EDTA separately weakly destroys the complex and affects the content of the metal ions in the complex [35]. EDTA effect on the SPC dissociation increases strongly in the presence of 8 M urea and 1 M NaCl [35]. This may indicate that the metal ions bound with proteins of SPC or trapped in cavities become readily available for EDTA only after the destruction of the hydrogen bonds and electrostatic contacts between the proteins of the stable complex.

In conclusion, in this work, the analysis of the relative content of sixteen elements in human milk and possible regularities of accumulation of these elements in oligomeric complexes of proteins was performed for the first time.

## Figures and Tables

**Table 1:** The comparison of the content of elements in the lyophilized preparations SPC_mix_ (from nine milk preparations) and lyophilized defatted milk preparations.

Element	Relative content, *μ*g/g		Ratio of values 1 and 2
	SPC (1)∗	Average for nine milk preparation (2)∗∗	
Ca	4100±137	3500±3368	1.2
P	2500±125	956.7±±444.0	2.6
Zn	340±14	35.5±27.3	9.6
Mg	260±10	217.8±117.7	1.2
Al	124±6	41.9±43.6	3.0
Fe	105±4	27.8±25.8	3.8
Cu	49±3	4.0±2.7	12.3
Ni	20±1	<0.53∗∗∗	>38
B	15±0.6	0.76±0.51	19.7
Sr	11±0.7	< 0.1	>110
Mn	4.1±0.2	< 0.05	> 82
Cr	3.1±0.1	< 0.5	>6.2
Ba	2.9±0.15	< 0.5	> 5.8
Pb	2.0±0.1	< 0.5	> 4.0
Cd	0.9±0.05	< 0.1	> 9.0
Ag	0.86±0.04	<0.03	> 28.7

∗Preparations of stable protein complexes (SPCs) from nine types of human milk were lyophilized and the relative content of different elements in SPC_mix_ (mixture of the complexes from nine milk preparations) was determined by two-jet plasma atomic emission spectrometry; the relative standard deviation of the values from three replicates was within 5-7 %.

∗∗Average concentration of elements in the case of nine milk lyophilized preparations (see Table 2).

∗∗The data of two-jet plasma atomic emission spectrometry contained reliable peaks corresponding to some elements, but it was possible to estimate only their approximate concentration.

**Table 2:** The relative content of elements in nine lyophilized preparations of human milk.

Element	Relative content, *μ*g/g of milk dried powder∗									Average value, *μ*g/g	Median, *μ*g/g	Interquartile ranges, *μ*g/g
	Number of milk preparation											
	1	2	3	4	5	6	7	8	9			
Ca	2200	500	2100	3700	4300	1800	2200	12000	2700	3500±3368	2200	3200
P	840	100	1000	1100	1700	770	830	1400	870	956.7±444.0	870	1000
Mg	110	50	290	280	330	150	210	410	130	217.8±117.7	210	240
Al	0.5	0.5	2.0	140	64	36	42	50	42	41.9±43.6	42	49.5
Zn	1.6	0.6	12.0	69	55	42	39	73	27	35.5±27.3	39	54.4
Fe	1.0	3.3	3.6	82	49	27	29	32	23	27.8±25.8	27	31
Cu	0.88	0.42	2.3	7.1	7.7	4.5	4.3	6.6	2.5	4.0±2.7	4.3	6.18
B	0.80	0.50	2.0	0.62	1.0	0.32	0.35	0.52	0.75	0.76±0.51	0.62	0.48
Ni	<0.5∗∗	<0.5	0.8	< 0.5	<0.5	<0.5	<0.5	<0.5	<0.5	<0.53	-	-
Ag	<0.03	<0.03	<0.03	<0.03	<0.03	<0.03	<0.03	<0.03	<0.03	<0.03	-	-
Sr	<0.1	<0.1	<0.1	<0.1	<0.1	<0.1	<0.1	<0.1	<0.1	<0.1	-	-
Ba	<0.5	<0.5	<0.5	<0.5	<0.5	<0.5	<0.5	<0.5	<0.5	<0.5	-	-
Pb	<0.5	<0.5	<0.5	<0.5	<0.5	<0.5	<0.5	<0.5	<0.5	<0.5	-	-
Cd	<0.1	<0.1	<0.1	<0.1	<0.1	<0.1	<0.1	<0.1	<0.1	<0.1	-	-
Cr	<0.5	<0.5	<0.5	<0.5	<0.5	<0.5	<0.5	<0.5	<0.5	<0.5	-	-
Mn	<0.05	<0.05	<0.05	<0.05	<0.05	<0.05	<0.05	<0.05	<0.05	<0.05	-	-

∗Preparations of 9 human milk were lyophilized and the relative content of different elements was determined by two-jet plasma atomic emission spectrometry; the relative standard deviation of the values from three replicates was within 5-7 %.∗∗The data of two-jet plasma atomic emission spectrometry contained reliable peaks corresponding to some elements, but the exact determination of their concentration was difficult.

**Table 3:** The comparison of the content of elements in the lyophilized milk preparation preparations and literature data.

Element	Relative content, mg/L∗			
	Milk (this article)∗∗	ICP-MS [38]∗∗∗	Method 1 [32]∗∗∗	Method 2 [32]∗∗∗
Ca	66.0-1584	36.7-375	8.4–688.9	0.90–12.7
Zn	0.08-9.6	0.03-3.8	0.07–0.56	0.05–0.26
Mg	6.6-54	-	1.0–18.7	0.67–4.22
Fe	0.13-10.8	0.71-1.85	0.10–12.3	0.28–7.2
Cu	0.06-1.0	55.6-419	0.06–0.17	<0.01–0.12
Mn	<0.007	0.22-30.3	<0.01–0.33	<0.01
Cr	<0.07		<0.01–0.86	<0.01
Pb	<0.07	0.21	2.5	-
P	13.2-224.4	-	-	-
Al	0.07-18.5	-	-	-
Ni	0.07-0.13	-	-	-
B	0.04-0.13	-	-	-
Sr	<0.013^ῼ^	-	-	-
Ba	<0.07		-	-
Cd	<0.013		-	-
Ag	<0.004		-	-

∗The content of various elements in the milk preparations; the ranges of values from the minimal to the maximal content are given (mg/L)

∗∗Human milk was lyophilized and the content of different elements was determined by two-jet plasma atomic emission spectrometry; the relative standard deviation in the case of every value from three replicates was within 5-7 %.

∗∗∗Comparison of our findings with the literature data: the concentration of elements was estimated using inductively coupled plasma mass spectrometry (ICP-MS) [38], 8-hydroxyquinoline approach (method 1) and ammonium pyrrolidine-dithiocarbamate as complexing agent (method 2) [32].

^ῼ^The data of two-jet plasma atomic emission spectrometry contained reliable peaks corresponding to some elements, but the exact determination of their concentration was difficult.

## Data Availability

The data used to support the findings of this study are available from the corresponding author upon request.

## Abbreviations

AAS: Atomic absorption spectrometry
HSA: Human serum albumin
ICP-AES: Inductively coupled plasma atomic emission spectrometry
ICP-MS: Inductively coupled plasma mass spectrometry
LS: Light scattering
TJP-AES: Two-jet plasma atomic emission spectrometry.
